# Epidemiology of zoonotic tick-borne diseases in Latin America: Are we just seeing the tip of the iceberg?

**DOI:** 10.12688/f1000research.17649.2

**Published:** 2019-02-11

**Authors:** Alfonso J. Rodriguez-Morales, D. Katterine Bonilla-Aldana, Samuel E. Idarraga-Bedoya, Juan J. Garcia-Bustos, Jaime A. Cardona-Ospina, Álvaro A. Faccini-Martínez

**Affiliations:** 1 Public Health and Infection Research Group, Faculty of Health Sciences, Universidad Tecnológica de Pereira, Pereira, Risaralda, 660003, Colombia; 2 School of Medicine, Universidad Franz Tamayo/UNIFRANZ, Cochabamba, Cochabamba, 4780, Bolivia; 3 Grupo de Investigación Sanidad Animal, Fundación Universitaria Autónoma de las Américas, Pereira, Risaralda, 660004, Colombia; 4 Grupo de Investigación en Patología e Inmunología – Doctorado en Medicina Tropical, Universidad del Magdalena, Santa Marta, Magdalena, 470004, Colombia; 5 Grupo de Investigación en Ciencias Animales Macagual, Universidad de La Amazonia, Florencia, Caquetá, 180002, Colombia; 6 Infection and Immunity Research Group, Faculty of Health Sciences, Universidad Tecnológica de Pereira, Pereira, Risaralda, 660003, Colombia; 7 Grupo de Investigación Biomedicina, Fundación Universitaria Autónoma de las Américas, Pereira, Risaralda, 660004, Colombia; 8 Emerging Infectious Diseases and Tropical Medicine Research Group, Instituto para la Investigación en Ciencias Biomédicas – Sci-Help, Pereira, Risaralda, 660003, Colombia; 9 Postgraduate Program in Infectious Diseases, Health Science Center, Universidade Federal do Espírito Santo, Vitória, ES, Brazil

**Keywords:** Tick-borne disease, zoonoses, Anaplasma, Babesia, Borrelia, Ehrlichia, Rickettsia, epidemiology, public health

## Abstract

Ticks are responsible for transmission of multiple bacterial, parasitic and viral diseases. Tick-borne diseases (TBDs) occur particularly in tropical and also subtropical areas. The frequency of these TBDs has been increasing and extending to new territories in a significant way, partly since ticks’ populations are highly favored by prevailing factors such as change in land use patterns, and climate change. Therefore, in order to obtain accurate estimates of mortality, premature mortality, and disability associated about TBDs, more molecular and epidemiological studies in different regions of the world, including Latin America, are required. In the case of this region, there is still a limited number of published studies. In addition, there is recently the emergence and discovering of pathogens not reported previously in this region but present in other areas of the world. In this article we discuss some studies and implications about TBDs in Latin America, most of them, zoonotic and with evolving taxonomical issues.

Over the past decades, there have been significant achievements in the understanding of tick-borne diseases (TBDs), which are mostly zoonoses and classed as neglected diseases ^1– 5^. Their occurrence is significant in tropical and subtropical areas, leading to an important impact on public health as well as the economy, as they affect humans, domestic animals, and livestock, among others ^6^. Knowledge of the occurrence of these diseases in animal species is of utmost importance for the understanding of the risk for human infection ^7^. Ticks, and animals, including human beings, interact with nature, and their environmental and ecological interactions regulate the populations of ticks and vertebrates, determining their contact rates and the circulation of the diseases ^8^. Regarding TBDs that affect humans, those that are caused by *Rickettsia* genera, known as spotted fever group rickettsioses, are the most studied and recognized in Latin America. Nevertheless, case reports and preliminary field studies published in the last two decades, suggest that ehrlichiosis, anaplasmosis, babesiosis and relapsing fever group borreliosis, would also be present but probably are underdiagnosed.

After the first description of *Rickettsia rickettsii* in North America in the first half of the twentieth century, this species also was recognized as a human pathogen in Latin America ^9^. Currently, *R. rickettsii* rickettsiosis is the most important and deadly TBDs in México, Panamá, Colombia, Brazil, and Argentina, where is transmitted to humans by different ticks’ species as *Rhipicephalus sanguineus*, *Amblyomma mixtum*, *A. patinoi*, *A. sculptum*, *A. aureolatum*, and *A. tonelliae* ^9, 10^. Unfortunately, only in Brazil, this disease is of officially mandatory reporting ^9^. Moreover, in the last years, other rickettsiae have been pointed as emerging pathogenic species, causing febrile rickettsiosis ( *R. parkeri* and *R. massiliae*) or asymptomatic/mild illness ( *R. amblyommatis*) ^9, 10^. Currently, *R. parkeri*, transmitted by *A. ovale*, *A. tigrinum*, and *A. triste*, is the main agent relate to eschar-associated rickettsiosis in Brazil, Argentina and Uruguay ^11– 13^. Clinically is less severe compared to *R. rickettsii* rickettsiosis, and no related deaths have been reported ^11– 13^.

On the other hand, although *Amblyomma americanum* and *Ixodes scapularis* ticks, which are recognized as main vectors of human pathogenic *Ehrlichia* and *Anaplasma* species in the United States, are not presented in Latin America ^14^, some confirmed *E. chaffeensis* infections have been reported in patients from Venezuela and Mexico ^15, 16^. The above suggests that probably other ticks’ species could be competent vectors in tropical regions. Thus, is worth to mention the recent descriptions of *Ehrlichia* spp. detected in anthropophilic ticks ( *A. tigrinum* and *A. parvum*) in Argentina ^17, 18^. Furthermore, particularly in Venezuela, few studies point *Anaplasma platys* and *E. canis* as a human pathogen ^19, 20^, a concern that actually discusses, but contrast with the recent description of a novel genotype of *E. canis* detected in samples of human blood bank donors in Costa Rica ^21^. The significance of the above requires future investigations.

Babesiosis is another tick-borne disease, caused by protozoal hemoparasites of the phylum Apicomplexa. Presently, three species of the genus *Babesia* ( *B. microti*, *B. divergens* and *B. venatorum*) are the main human pathogens in The United States, Europe, and Asia, where anthropophilic ticks of the *Ixodes ricinus* complex ( *I. scapularis*, *I. ricinus*, and *I. persulcatus*) are the main vectors ^22^. In Latin America, these tick’s species are not present, and even though exist some species of the *I. ricinus* complex, they do not human-biting ^14^. Nevertheless, interestingly, some confirmed *B. microti* infection has been reported in Mexico and Bolivia ^23, 24^, and also in the latter and in Colombia serological studies suggest exposure to *Babesia* spp. in rural individuals ^23, 25^. Acarological studies attempting to detect *Babesia* species in anthropophilic Latin American ticks are scarce.

Additionally, as occurs with Babesiosis, human-biting *I. ricinus* complex ticks are also vectors of pathogenic- *Borrelia burgdorferi* sensu lato (s.l.) species ( *B. burgdorferi* sensu stricto, *B. mayonii*, *B. garinii*, *B. afzelii*), causing Lyme borreliosis in temperate regions of northern hemisphere ^26^. In Latin America, in the last decade, new *B. burgdorferi* s.l. strains or new related species have been described in countries such as Argentina, Uruguay, Brazil, and Chile, from non-anthropophilic *Ixodes* tick ^27^. This fact, as well as that *B. burgdorferi*, has not yet been isolated or cultured from clinical samples from autochthonous patients, is against of Lyme borreliosis presence in Southern hemisphere of America. By contrast, considering the recently first isolation and molecular characterization of a relapsing fever *Borrelia* ( *B. venezuelensis*) in Latin America, recovered from an *Ornithodoros rudis* tick ^28^, is plausible the occurrence of underdiagnosed human cases, taking to account the historical records of tick-borne relapsing fever in Colombia, Venezuela and Panama ^29^.

Beyond the Americas, in other regions of the world, like in Europe, ticks are the main vectors of animal and human organisms. Ticks transmit several viral agents, called tick-borne viruses (TBV), such as tick-borne encephalitis virus and Crimean-Congo hemorrhagic fever virus, which have reemerged in multiple areas of the world ^30^. TBV have a natural cycle between ticks and wild animals in nature, with humans as accidental hosts ^30, 31^. Emerging TBVs are continually discovered, probably related to the increase of tick populations in different regions of the planet and invasion of human beings into areas infested by ticks ^30, 31^. The study of tick-borne viruses in Latin America is scarce. Recently Brazilian authors described a genetic characterization of Cacipacoré virus (genus Flavivirus) from *A. cajennense* ticks collected in São Paulo State, Brazil ^32^. The significance of this finding requires future investigations.

**Table 1.** Examples of selected tick-borne diseases in Latin America.

Disease	Etiological agent(s)	Primary or probable vector(s)
*Rickettsia rickettsii* rickettsiosis	*Rickettsia rickettsii*	*Rhipicephalus sanguineus*, *Amblyomma mixtum*, *A. patinoi*, *A. sculptum*, *A. aureolatum*, *A. tonelliae*
*Rickettsia parkeri* rickettsiosis	*Rickettsia parkeri*	*A. ovale*, *A. maculatum*, *A. tigrinum, A. aureolatum, A. triste*
*Rickettsia massiliae* rickettsiosis	*Rickettsia massiliae*	*Rhipicephalus sanguineus*
Ehrlichiosis ?	*Ehrlichia* sp. strain San Luis and strain Cordoba	*A. tigrinum*, *A. parvum*
Tick-borne relapsing fever	*Borrelia venezuelensis*	*Ornithodoros rudis*

Detection and sentinel surveillance of TBDs require molecular tools for diagnosis ^33^, for example, serological tests have proven to be inconclusive in diagnose Lyme disease ^34^. The use of molecular biology tests in recent years has increased the sensitivity and specificity of the diagnosis of infections caused by Rickettsiales. Molecular diagnosis enables the accurate identification not only at the genus level but species, providing additional characterization on the epidemiology and the evolution of the clinical disease. Furthermore, PCR, as well as enzyme restriction tests of the vector blood meal, can be employed to analyze their feeding source and possibly identify the ecological reservoir of the organisms ^35^.

## Conclusions

Besides the number of studies in Latin America on TBDs, the prevalence of these diseases is increasing, triggered by globalization, as well the impact climate change and variability. More surveillance, more diagnostics, with better identification approaches, as well as more research, is needed. Even more, in that way, there is a lack of infrastructure and/or funding to support continued vector surveillance studies in many countries across the region. Tick and TBDs investigators, veterinary doctors, medical and public health practitioners should work to share their expertise on different aspects of TBDs, such as tick ecology, disease transmission, diagnostics, and treatment, in order to face the challenges of scientific, political, and public engagement for TBD research and control in this region ^36^. Systematic reviews, as well as observational analyses, are necessary in order to understand the current situation of TBDs. In fact, also there is a lack of studies of costs and burden of these diseases, as is clearly available for other vector-borne diseases (e.g. arboviral) ^37^. As is known, also there are clear limitations in the national budgets that are specifically earmarked for vector-borne surveillance and public health efforts. Even more, what part of that is allotted toward TBD research. This should be considered as part of this call to action. For diagnostics, molecular tools can provide valuable information for understanding the evolution of their etiological agents, as well as provide insights into host-pathogen-vector-environment interactions but need to be more widely available as part of routine diagnostics. Probably, what we have seen till now in terms of prevalence, but also in terms of action to reduce the impact of TBD, is just the tip of an iceberg and there is a need for more studies and actions towards control in Latin America about these diseases.

## Data availability

The data referenced by this article are under copyright with the following copyright statement: Copyright: © 2019 Rodriguez-Morales AJ et al.

Data associated with the article are available under the terms of the Creative Commons Zero "No rights reserved" data waiver (CC0 1.0 Public domain dedication). 

No data is associated with this article.
