# Supplementary material for: Epidemiology of zoonotic tick-borne diseases in Latin America: Are we just seeing the tip of the iceberg?
Source: F1000Res. 2019 Feb 11;7:1988. Originally published 2018 Dec 31. [Version 2] doi: 10.12688/f1000research.17649.2 (PMC6707394; doi:10.12688/f1000research.17649.2)
Supplement: Peer review history

# Peer review: Reviewer response for version 2

**DOI:** 10.5256/f1000research.19787.r52133

**Published:** 2019-08-19

**Authors:** Stefan Vilges de Oliveira

**Affiliations:** 1 Faculty of Medicine, Federal University of Uberlandia, Uberlândia, Brazil

I would like to highlight some points that could be improved:

 The title of the manuscript creates an expectation that is not answered. In the manuscript is finished stating that further studies could help answer this question. Methodologically this is not correct. Suggestion to change the title to: Epidemiology of zoonotic tick-borne diseases in Latin América.

 The authors mention that Brazil is the only country in Latin America that TBDs are mandatory reporting. In Brazil Spotted Fever is diseases the mandatory notification. Authors need to provide the correct source of information. They should mention the ordinance that regulates the notification in Brazil and that is available at this Link.

 About the title question: Are we just seeing the tip of the iceberg?

 Even in Brazil with the best data compared to other Latin American countries it was still not possible to answer this question.

 In order to measure these subrecords, it would be advisable to evaluate different sources of information through retrospective studies that include information on patients with clinical suspicions with differential diagnoses for TBDs.

 In Brazil different information systems are used for different functionalities. There is a system that records the notification diseases (Notification Disease Information System), another records the deaths (Mortality Information System), another the laboratory diagnosis requests (Laboratory Environment Manager). To evaluate subrecords it would be interesting to crosscheck data that takes into account the information from the different information systems. Ideally a system of clinical suspicion and laboratory investigation based on a syndromic diagnosis would be the most effective for detecting TBDs cases.

 Finally, the updated review highlights very important points, such as the lack of information, the need to assess the burden and impact of TBDs and the advances made so far and the challenges, especially in diagnosis, clinical and laboratory. Therefore, I consider this article appropriate for indexing.

I confirm that I have read this submission and believe that I have an appropriate level of expertise to confirm that it is of an acceptable scientific standard.

# Peer review: Reviewer response for version 2

**DOI:** 10.5256/f1000research.19787.r44291

**Published:** 2019-03-04

**Authors:** Joyce M Sakamoto

**Affiliations:** 1 Department of Entomology, The Pennsylvania State University, University Park, PA, USA

This second version of the manuscript entitled “Epidemiology of zoonotic tick-borne diseases in Latin America: Are we just seeing the tip of the iceberg?” has undergone edits, the inclusion of more references, and the addition of an author. I have chosen "Approved" because, although there are some minor comments that should be addressed, there is no "minor revision" option available. 

 Many of the edits that the authors have included have improved readability and flow. However, there are some points that need clarification. Most of these are grammatical/typographical errors, but a few have to do with structural organization of sentences or lack of citations. I would like to point out that, while it is commendable that the authors utilized a tool like Grammarly, the general (i.e. non-academic) version of Grammarly does not work as well for scientific terminology or syntax:

**Paragraph 1**: “Regarding TBDs that affect humans, those that are caused by *Rickettsia* genera, known as spotted fever group rickettsioses, are the most studied and recognized in Latin America."

 1) The wording is a little confusing because of the structure. Start with "The most important tick-borne diseases in Latin America are caused by pathogen spotted fever group *Rickettsia.*"

 2) Replace “genera". It should be “Multiple species in the genus *Rickettsia*” or “ *Rickettsia* spp.” There is only one genus of *Rickettsia.*

**Paragraph 2**: “Currently, *R. rickettsii* rickettsiosis is the most important and deadly TBDs in México, Panamá, Colombia, Brazil, and Argentina, where is transmitted to humans by different ticks’ species as *Rhipicephalus sanguineus, Amblyomma mixtum, A. patinoi, A. sculptum, A. aureolatum, and A. tonelliae*”

 1) Change “where”. It should be “which”.

 2) Change “ticks’ species as’” to “tick species such as”.

 3) Use "Rocky Mountain Spotted Fever" to describe the disease caused by *R. rickettsii. e.g. "*Currently, Rocky Mountain Spotted fever ( *R. rickettsii*) is the most important and deadly TBD..."

**Paragraph 3**: “Furthermore, particularly in Venezuela, few studies point *Anaplasma platys* and *E. canis* as a human pathogen, a concern that actually discusses, but contrast with the recent description of a novel genotype of *E. canis* detected in samples of human blood bank donors in Costa Rica”

 1) I am not sure I understand the point of this sentence. Grammatically, it seems like you are missing some words in this part of the sentence: “…few studies point *Anaplasma platys* and *E. canis* as a human pathogen…” should be changed to:

  “…studies point to *Anaplasma platys* and *E. canis* as human pathogen s…”?

 2) The *E. canis* genotype paper does not "contrast" with the human blood bank donor study. Blood tested from two case studies is a pretty low sample size (N = 2) to make any conclusion. Maybe just stop after " *E. canis* as human pathogens"

**Paragraph 5**: "This fact, as well as that *B. burgdorferi*, has not yet been isolated or cultured from clinical samples from autochthonous patients, is against of Lyme borreliosis presence in Southern hemisphere of America. “

 The structural organization of this sentence is confusing. Maybe: “Because *B. burgdorferi* s.l. has not been isolated or cultured from...It is not thought to be  present in…”

**Paragraph 5**: “ By contrast, considering the recently first isolation and molecular characterization of a relapsing fever *Borrelia* ( *B. venezuelensis*) in Latin America, recovered from an *Ornithodoros rudis* tick, is plausible the occurrence of underdiagnosed human cases, taking to account the historical records of tick-borne relapsing fever in Colombia, Venezuela and Panama. ”

 Organize this sentence differently. Break this sentence into two, e.g.: “In contrast, tick-borne relapsing fevers have historically occurred in Colombia, Venezuela, and Panama. Recently, the relapsing fever pathogen *Borrelia venezuelensis* has been isolated and molecularly characterized from the soft tick *Ornithodoros rudis*."  

**Paragraph 7**: "Detection and sentinel surveillance of TBDs require molecular tools for diagnosis, for example, serological tests have proven to be inconclusive in diagnose Lyme disease”

 There are a couple of things wrong with this sentence. 

 1) "diagnose" should be "diagnosis of" or "diagnostics of"

 2)  I am not sure serological tests are inconclusive, although there are problems with cross-reactivity or poor sensitivity during early Lyme disease onset ^1^.   

**Paragraph 7**  “… at the genus level but species, …. 

 • Change to:  “to the species level”

**Conclusion**: “As is known, also there are clear limitations in the national budgets that are specifically earmarked for vector-borne surveillance and public health efforts.Even more, what part of that is allotted toward TBD research. This should be considered as part of this call to action."

 The point of your piece is that there is a growing tick-borne disease problem that needs to be and is currently not addressed. “As is known” is referring to anecdotal information. Please find a reference to support this claim that national budgets are not providing enough funds for vector-borne surveillance and compare (or contrast) this with what you think it should be (probably the Colombian National Public Health Institutions will have information on this).

I confirm that I have read this submission and believe that I have an appropriate level of expertise to confirm that it is of an acceptable scientific standard.

## References

[1] : The Accuracy of Diagnostic Tests for Lyme Disease in Humans, A Systematic Review and Meta-Analysis of North American Research. *PLoS One*.2016;11(12) : 10.1371/journal.pone.0168613 e0168613 10.1371/journal.pone.0168613 28002488PMC5176185

# Peer review: Reviewer response for version 1

**DOI:** 10.5256/f1000research.19300.r42313

**Published:** 2019-01-30

**Authors:** Joyce M Sakamoto

**Affiliations:** 1 Department of Entomology, The Pennsylvania State University, University Park, PA, USA

This manuscript entitled "Epidemiology of zoonotic tick-borne diseases in Latin America: Are we just seeing the tip of the iceberg?" suggests that more studies on TTBD (ticks and tick-borne diseases) are needed worldwide, and particularly in Latin America, but there is a dearth of available data published. 

 Because the authors emphasized that their manuscript was not intended to be an extensive review article, but was originally presented as an opinion piece, I reviewed this manuscript as the latter. There is a lack of cohesiveness and transition between subjects within the manuscript and an overall lack of a common theme. There are also some minor errors in terminology and italicization throughout, which I will point out in each paragraph. In summary, while I appreciate the fact that this paper was originally intended to be a superficial review intended to emphasize the lack of research literature on ticks and TBD in Latin America, it does not do so in a cohesive manner, nor does it make a clear case for the need for further resources. Nevertheless, many Latin American countries would benefit from more resources dedicated to TBD research and surveillance. Should the authors reorganize and focus their attention to a specific objective, this might make for a stronger case. 

**Specific Comments:**

 The 1st paragraph gives a brief introduction to the impacts of TTBD on human and animal health, particularly in tropical and subtropical areas of the world. While it is interesting to point out that some TBD can be transmitted in other ways besides via ticks, I'm not sure that really supports the point of your manuscript. Make sure to be consistent in whether you use "tick-borne" or "tick borne" throughout the manuscript.

 The 2nd paragraph begins with an introduction to Latin America, but then goes directly into rickettsioses, followed by a paragraph each on ehrlichioses, babesioses, and a brief mention of borrelioses. This feels much like a listing of diseases that have no connectivity with the overall purpose of the article. To help this, a sentence or two to introduce what will be discussed in the coming paragraphs might help to outline what a reader might expect to see. Maybe something like…”Here we will briefly review the known literature and highlight the increasing incidence/discovery/etc of tick-borne pathogens…”

- There seems to be a missing word in " *Rickettsia* *rickettsii* were reported in rural and urban of Panama"
- "Until some years ago, R. rickettsia was the only tick-borne species of rickettsia present in Latin America.  Nowadays" - 'Nowadays' is too colloquial. Perhaps "Presently" or "Currently" are better alternatives? 
- There is what looks like an autocorrect error (" *R. rickettsia*" should be " *R.* *rickettsii*").
- Question: Is it possible that other *Rickettsia* spp. were present already, but the older serological diagnostics that identified RMSF ( *R. rickettsii*) were cross-reacting with them and were all recorded as RMSF?
- Many *Rickettsia *spp. are non-pathogenic and are instead obligate symbionts of the ticks in which they reside.

3rd paragraph: "specie" should be "species". The genus "Ehrlichia" should be italicized.

 4th paragraph: I refer you to "Rickettsia species". *Rickettsia *capitalized refers to the genus and it should be italicized. If referring to the colloquial term used to refer to Rickettsiales, then it should be lowercase and not italicized (rickettsias). I believe the former applies in this sentence. "sp." indicates that the species designation is not known and is not italicized.

 5th paragraph:

- "Till" should be "Until".
- The paragraph is almost entirely about babesioses worldwide, yet the last sentence briefly mentions *Borrelia *and does not flow at all. Borreliosis should be its own section, with a discussion on both Lyme borreliosis and relapsing fever variants. Perhaps the authors could mention the role that soft ticks play in relapsing fever, particularly since this is a problem in Latin America.

6th paragraph:

- If the purpose of this paper is to focus on Latin America and the paucity of work on ticks and tickborne disease relative to other parts of the world, there should be more focus on Latin America. The discussion of other countries detracts from this message. The only exception is in the context of potential TTBD flow between these countries via trade, human or animal migration, and impacts of climate change.
- Further, there is a sudden switch mid-paragraph to tickborne viruses (TBV). Is the purpose of mentioning TBV to say that TBV exist in Latin America, but no one has looked hard enough? There are a few review articles that may provide support for this, but I suggest that as it is here, there is no context and it feels like it was just added after-the-fact. 
- Replace “vectorized” with “transmitted”. Note “Transmit” = verb, “vector” = noun. "Vectorized” isn’t really a word, or at least not commonly used North American medical entomology.

7th paragraph: I'm unclear on the purpose of this paragraph. Is it to highlight the advances in diagnostics that make it possible to detect TBD? If so, how does this support the overall theme of this article?

- The last sentence of the 7th paragraph is not clear and has several errors. "Etiological agents of the group of **Rickettsial**, including those in the **genuses** *Anaplasma*, *Neorickettsia*, *Ehrlichia*, and *Rickettsia*, are relevant and often vector-borne organisms of canines and felines, but also of bovine, live-stock and other animals, which appears to be a wide range of hosts". Could this be moved somewhere else to make a transition or taken out completely?
- “group of “Rickettsial” should be either “rickettsial pathogens in the…” OR  “Etiological agents in the Order Rickettsiales”
- "live-stock" should be "livestock"
- “Genuses” should be “genera"
- The Order Rickettsiales is an Order and not italicized. Admittedly this order's taxonomy is problematic, but if you are going to refer to it, don't italicize it.

Table 1:

- Your table is entitled “Examples of selected tick-borne diseases in Latin America,” yet contains tick species mostly localized to North America ( *D.* *andersoni* *, D.* *variabilis* *, D.* *occidentalis* *, I.* *pacificus* *, *and *I.* *scapularis*). There was a review by Esteve-Gassent et al (2014) ^1^ that suggests that some of these species could potentially spill over the Mexico-USA border and therefore these species could potentially warrant further study. Your use of this table, however, does not provide any context and feels out-of-place and irrelevant.
- There have been several articles detailing known hard and soft tick species and their epidemiological significance from many different Latin American countries (e.g. Rivera-Páez in 2018 gives updates to Colombian Ixodidae ^2^; Mastropaolo 2014 reviewed both hard and soft ticks of Bolivia ^3^; Lopes in 2016 of Belize ^4^, and Witter in 2016 from wild animals of Brazil ^5^, just to name a few). There have also been reviews on tick species found in the Caribbean, Cuba, and Mexico. If you can obtain it, a valuable and comprehensive resource is “The Hard Ticks of the World (Acari: Ixodida: Ixodidae)”, by Guglielmone et al (2014) ^6^, which contains summaries of all known hard tick species worldwide, including host associations and geographic distributions. 

Conclusion: I am afraid I don't see the relevance of this conclusion section to the rest of the article. What you need is to tie together what you have written and make a conclusion. 

- What is your conclusion? Do we need more surveillance? More research? Better diagnostics? Better identification approaches?
- What are you trying to state that leads to your final concluding statement that this is "just the tip of the iceberg"? To say there is not enough data is sort of vague. More data is always better, but I myself have at least 100 references for literature on TTBD from Central and South America, and my list is not extensive. I would posit that you may need to rethink the purpose of your article. To state that there insufficient work on this topic in Latin America borders on insulting those who have spent careers working on these exact topics. 
- There is an element that you have not discussed: the lack of infrastructure and/or funding to support continued vector surveillance studies. Are there reports comparing the estimated costs of these types of studies (surveillance as well as diagnostics)? If so, how does that compare to the estimated proportion of the national budgets that are specifically earmarked for vector-borne surveillance and public health efforts, and what part of that is allotted toward TTBD research? Perhaps this would strengthen your case for a call to action.

I confirm that I have read this submission and believe that I have an appropriate level of expertise to confirm that it is of an acceptable scientific standard, however I have significant reservations, as outlined above.

## References

[1] : Pathogenic Landscape of Transboundary Zoonotic Diseases in the Mexico-US Border Along the Rio Grande. *Front Public Health*.2014;2: 10.3389/fpubh.2014.00177 177 10.3389/fpubh.2014.00177 25453027PMC4233934

[2] : Contributions to the knowledge of hard ticks (Acari: Ixodidae) in Colombia. *Ticks Tick Borne Dis*.9(1) : 10.1016/j.ttbdis.2017.10.008 57-66 10.1016/j.ttbdis.2017.10.008 29055642

[3] : The ticks (Acari: Ixodida: Argasidae, Ixodidae) of Bolivia. *Ticks Tick Borne Dis*.2014;5(2) : 10.1016/j.ttbdis.2013.10.005 186-94 10.1016/j.ttbdis.2013.10.005 24406053

[4] : Ticks and rickettsiae from wildlife in Belize, Central America. *Parasit Vectors*.2016;9: 10.1186/s13071-016-1348-1 62 10.1186/s13071-016-1348-1 26831147PMC4736261

[5] : Rickettsial infection in ticks (Acari: Ixodidae) of wild animals in midwestern Brazil. *Ticks Tick Borne Dis*.2016;7(3) : 10.1016/j.ttbdis.2015.12.019 415-23 10.1016/j.ttbdis.2015.12.019 26775021

[6] : The Hard Ticks of the World.2014; 10.1007/978-94-007-7497-1 10.1007/978-94-007-7497-1

# Response

**Published:** 2019-02-04

**Authors:** Alfonso Rodriguez-Morales

**Affiliations:** Universidad Tecnológica de Pereira, Colombia

Dear Dr. Sakamoto

Thanks for your valuable comments. We want to answer all your comments, as we improved significantly our manuscript based on yours as well as on the other reviewer.

**This manuscript entitled "Epidemiology of zoonotic tick-borne diseases in Latin America: Are we just seeing the tip of the iceberg?" suggests that more studies on TTBD (ticks and tick-borne diseases) are needed worldwide, and particularly in Latin America, but there is a dearth of available data published.**

Certainly, there are more available data published, but our original intention was present an Opinion Article. As an Advisor of this Gateway, called, Disease Outbreaks, I should explain to you that at this open publishing platform where there are Opinion Articles, Review Articles and Systematic Review Articles.

This article is NOT a Systematic Review, it was originally submitted as an Opinion Article, as an invitation from F1000Research as being F1000Research Disease Outbreaks Gateway Advisor, but later classified as Review (narrative review). As you well read, we only referred to some examples of studies in Latin America, including yours (cited, now 36. Sakamoto JM: Progress, challenges, and the role of public engagement to improve tick-borne disease literacy. Curr Opin Insect Sci. 2018;28:81–9. 30551772 10.1016/j.cois.2018.05.011), about tick-borne diseases, that illustrate the problem, in terms of a neglected group of conditions in the region, wherein most of the countries are not under surveillance, and there is still a lack of studies, but even more actions for effective control.

**Because the authors emphasized that their manuscript was not intended to be an extensive review article, but was originally presented as an opinion piece, I reviewed this manuscript as the latter. There is a lack of cohesiveness and transition between subjects within the manuscript and an overall lack of a common theme. There are also some minor errors in terminology and italicization throughout, which I will point out in each paragraph. In summary, while I appreciate the fact that this paper was originally intended to be a superficial review intended to emphasize the lack of research literature on ticks and TBD in Latin America, it does not do so in a cohesive manner, nor does it make a clear case for the need for further resources. Nevertheless, many Latin American countries would benefit from more resources dedicated to TBD research and surveillance. Should the authors reorganize and focus their attention to a specific objective, this might make for a stronger case.** 

We have significantly improved the grammatical and orthography of this paper. We modified the paper considering your comments.

**Specific Comments:**

**The 1st paragraph gives a brief introduction to the impacts of TTBD on human and animal health, particularly in tropical and subtropical areas of the world. While it is interesting to point out that some TBD can be transmitted in other ways besides via ticks, I'm not sure that really supports the point of your manuscript. Make sure to be consistent in whether you use "tick-borne" or "tick borne" throughout the manuscript.**

Well, that's a comment, additionally to the main point of this article. Regard your comment of consistency in the use of tick-borne, we have checked and only left "tick-borne".

**The 2nd paragraph begins with an introduction to Latin America but then goes directly into rickettsioses, followed by a paragraph each on ehrlichioses, babesioses, and a brief mention of borrelioses. This feels much like a listing of diseases that have no connectivity with the overall purpose of the article. To help this, a sentence or two to introduce what will be discussed in the coming paragraphs might help to outline what a reader might expect to see. Maybe something like…”Here we will briefly review the known literature and highlight the increasing incidence/discovery/etc of tick-borne pathogens…”**

We have restructured the paragraph in that way.

 

- **There seems to be a missing word in " *Rickettsia*** ***rickettsii*** **were reported in rural and urban of Panama". **Corrected.
- **"Until some years ago, R. rickettsia was the only tick-borne species of rickettsia present in Latin America.  Nowadays" - 'Nowadays' is too colloquial. Perhaps "Presently" or "Currently" are better alternatives?** Corrected.
- **There is what looks like an autocorrect error (" *R. rickettsia*" should be " *R.*** ***rickettsii*** **")**. Corrected.
- **Question: Is it possible that other *Rickettsia* spp. were present already, but the older serological diagnostics that identified RMSF ( *R.*** ***rickettsii*** **) were cross-reacting with them and were all recorded as RMSF?** Included.
- **Many *Rickettsia *spp. are non-pathogenic and are instead obligate symbionts of the ticks in which they reside.** Considered.

**3rd paragraph: "specie" should be "species". The genus "Ehrlichia" should be italicized.**

Done. All that should be italicized, has been done.

**4th paragraph: I refer you to "Rickettsia species". *Rickettsia *capitalized refers to the genus and it should be italicized. If referring to the colloquial term used to refer to Rickettsiales, then it should be lowercase and not italicized (rickettsias). I believe the former applies in this sentence. "sp." indicates that the species designation is not known and is not italicized**.

Done. Capitalized where corresponded.

5th paragraph:

- **"Till" should be "Until".** Ok.
- **The paragraph is almost entirely about** **babesioses** **worldwide, yet the last sentence briefly mentions *Borrelia *and does not flow at all. Borreliosis should be its own section, with a discussion on both Lyme borreliosis and relapsing fever variants. Perhaps the authors could mention the role that soft ticks play in relapsing fever, particularly since this is a problem in Latin America.** This has been modified. We discussed also relapsing fever.

6th paragraph:

- **If the purpose of this paper is to focus on Latin America and the paucity of work on ticks and** **tickborne** **disease relative to other parts of the world, there should be more focus on Latin America. The discussion of other countries detracts from this message. The only exception is in the context of potential TTBD flow between these countries via trade, human or animal migration, and** **impacts** **of climate change. **We have improved this aspect and more focused on Latin America.
- **Further, there is a sudden switch mid-paragraph to tickborne viruses (TBV). Is the purpose of mentioning TBV to say that TBV exist in Latin America, but no one has looked hard enough? There are a few review articles that may provide support for this, but I suggest that as it is here, there is no context and it feels like it was just added after-the-fact.** We have corrected this.
- **Replace “vectorized” with “transmitted”. Note “Transmit” = verb, “vector” = noun. "Vectorized” isn’t really a word, or at least not commonly used North American medical entomology.** Corrected.

**7th paragraph: I'm unclear on the purpose of this paragraph. Is it to highlight the advances in diagnostics that make it possible to detect TBD? If so, how does this support the overall theme of this article? **We restructured the paragraph.

- **The last sentence of the 7th paragraph is not clear and has several errors. "Etiological agents of the group of Rickettsial, including those in the **genuses***Anaplasma*, *Neorickettsia*, *Ehrlichia*, and *Rickettsia*, are relevant and often vector-borne organisms of canines and felines, but also of bovine,** **live-stock** **and other animals, which appears to be a wide range of hosts". Could this be moved somewhere else to make a transition or taken out completely?** Corrected.
- **“group of “Rickettsial” should be either “rickettsial pathogens in the…” OR  “Etiological agents in the Order Rickettsiales”.** Corrected.
- **"live-stock" should be "livestock".** Done.
- **“** **Genuses** **” should be “** **genera** **".** Done.
- **The Order Rickettsiales is an Order and not italicized. Admittedly this order's taxonomy is problematic, but if you are going to refer to it, don't italicize it.** Ok.

Table 1:

- **Your table is entitled “Examples of selected tick-borne diseases in Latin America,” yet contains tick species mostly localized to North America ( *D.*** ***andersoni*** ***, D.*** ***variabilis*** ***, D.*** ***occidentalis*** ***, I.*** ***pacificus*** ***, *and *I.*** ***scapularis*** **). There was a review by Esteve-Gassent et al (2014) ^1^ that suggests that some of these species could potentially spill over the Mexico-USA border and therefore these species could potentially warrant further study. Your use of this table, however, does not provide any context and feels out-of-place and irrelevant**. We have extensively changed the table.
- **There have been several articles detailing known hard and soft tick species and their epidemiological significance from many different Latin American countries (e.g. Rivera-Páez in 2018 gives updates to Colombian Ixodidae ^2^; Mastropaolo 2014 reviewed both hard and soft ticks of Bolivia ^3^; Lopes in 2016 of Belize ^4^, and Witter in 2016 from wild animals of Brazil ^5^, just to name a few). There have also been reviews on tick species found in the Caribbean, Cuba, and Mexico. If you can obtain it, a valuable and comprehensive resource is “The Hard Ticks of the World (Acari: Ixodida: Ixodidae)”, by Guglielmone et al (2014) ^6^, which contains summaries of all known hard tick species worldwide, including host associations and geographic distributions.** We have corrected that.

**Conclusion: I am afraid I don't see the relevance of this conclusion section to the rest of the article. What you need is to tie together what you have written and make a conclusion. **

- **What is your conclusion? Do we need more surveillance? More research? Better diagnostics? Better identification approaches?** We included those considerations.
- **What are you trying to state that leads to your final concluding statement that this is "just the tip of the iceberg"? To say there is not enough data is sort of vague. More data is always better, but I myself have at least 100 references for literature on TTBD from Central and South America, and my list is not extensive. I would posit that you may need to rethink the purpose of your article. To state that there insufficient work on this topic in Latin America borders on insulting those who have spent careers working on these exact topics**. Now we have considered that and discussed in the Conclusions.
- **There is an element that you have not discussed: the lack of infrastructure and/or funding to support continued vector surveillance studies. Are there reports comparing the estimated costs of these types of studies (surveillance as well as diagnostics)? If so, how does that compare to the estimated proportion of the national budgets that are specifically earmarked for vector-borne surveillance and public health efforts, and what part of that is allotted toward TTBD research? Perhaps this would strengthen your case for a call to action.** We mentioned all of that now and tried to make a more deep call for action.

# Peer review: Reviewer response for version 1

**DOI:** 10.5256/f1000research.19300.r42312

**Published:** 2019-01-30

**Authors:** Lidia Chitimia-Dobler

**Affiliations:** 1 Bundeswehr Institute of Microbiology, Munich, Germany

Title and content of this work do not fit together.

 What about the taxonomical issues stated at the end of the abstract? It is not discussed in the paper.

 For a review, it is not very comprehensive and does not focus on Latin America. The structure of the manuscript is not logical, starting with *Rickettsia*, continue with *Ehrlichia*, coming back to *Rickettsia*. There is too much content about the USA for a short review referring to Latin America.

 In the table, there are a number of tick-borne diseases which do not occur in Latin America E.g. Colorado tick fever, Heartland virus diseases or Powassan encephalitis. It is not acceptable to simply transfer data from USA to Latin America.

I confirm that I have read this submission and believe that I have an appropriate level of expertise to state that I do not consider it to be of an acceptable scientific standard, for reasons outlined above.

# Response

**Published:** 2019-02-04

**Authors:** Alfonso Rodriguez-Morales

**Affiliations:** Universidad Tecnológica de Pereira, Colombia

Dear Dr. Chitima-Dobler

Thanks for your valuable comments. We want to answer all your comments, as we improved significantly our manuscript based on yours as well as on the other reviewer.

**Title and content of this work do not fit together.**

Well, we have significantly improved the manuscript, which was originally intended as an Opinion Article. As an Advisor of this Gateway, called, Disease Outbreaks, I should explain to you that at this open publishing platform where there are Opinion Articles, Review Articles and Systematic Review Articles.

This article is NOT a Systematic Review, it was originally submitted as an Opinion Article, as an invitation from F1000Research as being F1000Research Disease Outbreaks Gateway Advisor, but later classified as Review (narrative review). As you well read, we only referred to some examples of studies in Latin America, about tick-borne diseases, that illustrate the problem, in terms of a neglected group of conditions in the region, wherein most of the countries are not under surveillance, and there is still a lack of studies, but even more actions for effective control.

**What about the taxonomical issues stated at the end of the abstract? It is not discussed in the paper.**

We have improved on the updated taxonomy.

**For a review, it is not very comprehensive and does not focus on Latin America. The structure of the manuscript is not logical, starting with *Rickettsia*, continue with *Ehrlichia*, coming back to *Rickettsia*. There is too much content about the USA for a short review referring to Latin America.**

As we mentioned, this is not a Systematic Review, is an opinion article, published by decision of F1000Research as a Review (narrative). We focused now more in Latin America.

**In the table, there are a number of tick-borne diseases which do not occur in Latin America E.g. Colorado tick fever, Heartland virus diseases or Powassan encephalitis. It is not acceptable to simply transfer data from** **USA** **to Latin America.**

We significantly changed and corrected the table.
